# The Nature of "Idiopathic" Epilepsy

**Published:** 1908-04-04

**Authors:** 


### The Hospital

A Journal of

**The Medical Sciences and Hospital Administration.**

New Series. No. 55, Vol. III. [No. 1127, Vol. XLIV.] SATURDAY, APRIL 4, 1908.

### The Nature of "Idiopathic" Epilepsy.

After having long been catalogued with the functional disturbances, "idiopathic" epilepsy came by more or less general consent to be reckoned an organic disorder. This, no doubt, resulted from the frequency with which organic lesions were found among inveterate epileptics who gravitated to asylums for the insane, and the want of a clinical criterion capable of effecting the distinction between the organic and idiopathic varieties led to the assumption of a permanent lesion in every instance. Such an assumption naturally gave the treatment [illegible]d prognosis of the disease a pessimistic cast, but [illegible] recent years a further study of the features of [illegible]epsy in its greatly varied associations has made opinion on the matter less dogmatic. The intimate connection not infrequently observed in childhood between some focal disturbance distant from the brain—such, for example, as follicular tonsillitis—and an epileptic seizure, especially when occurring in a subject not previously liable to the latter malady, can hardly be reconciled with any but a functional basis for the particular seizure concerned. It is not at all uncommon to meet with children who suffer from epileptic manifestations at intervals of time so protracted that one cannot conceive them to depend upon an enduring structural alteration of the cortex cerebri. Again, even in those subject to epilepsy the influence of mental agitation in determining an attack is highly suggestive of a functional basis for the phenomenon. For example, a man applied one night for admission to a hospital on the ground that he was an epileptic subject and knew he was about to have a fit. This foreknowledge he attributed to an indescribable aura situated below the knees. As the hour was very late, he was suspected of malingering for a night's lodging. In response to inquiries he stated that he had spent the night in gambling, had lost much more than he could afford, and was afraid to go home under such circumstances and at such an hour. His obvious agitation merely served to confirm the suspicions already aroused as to the nature of his application, but while a prescription was being written for him he fell into a typical epileptic attack of whose genuineness there could be no question.

The consideration of cases such as these leaves no escape from the conclusion that epileptic phenomena may be symptomatic of functional disorder pure and simple—a conclusion of the most hopeful significance. Corroboration of this optimistic view has lately been offered by Dr. J. A. McCallum,* who speaks from a large experience gained while in charge of an epileptic colony for boys. His treatment, which consists in the administration of bromides in phenomenal doses, is based "on the assumption that early epilepsy is far more frequently the direct result of peripheral irritation than of Gowers's 'abnormal state of chemical nutrition,' and that the liberal use of bromide prevents this peripheral reflex from disturbing the cerebral harmony, and saves the brain from contracting the bad habit of epilepsy on the slightest sensory disturbances. The dose of bromide given is that amount which is necessary to control the fits. This is done in some cases by 40 grains a day: oftener 100 grains are required, and as much as 300 grains has been required." The practical application of Dr. McCallum's conclusion has yielded results which are extremely encouraging. Every physician is familiar with the sense of helplessness produced by the apparent failure of bromide treatment in a recalcitrant case of epilepsy: for the recognised second line of attack, consisting of belladonna, borax, dietetic regulations, and the like, has not done much to inspire confidence. It remains to be seen whether in other hands the "radical" treatment by bromides advocated by Dr. McCallum will come up to the expectations it raises, but it is something to have a new weapon in our armoury which, at the least, does no harm. This statement may appear to be hasty, in view of the immense doses recommended, but the proof of the pudding is in the eating, and the facts seem clear enough. We are told that epileptic boys stand the bromides very well, being able to take as much as a hundred grains a day without showing any signs that they are being drugged. Increase of the dose beyond this point is prone to produce disturbances of equilibrium, which demand that the treatment should be carried out in bed. Bromide eruptions receive very cavalier notice from Dr. McCallum, who assures us that they may well be ignored if the best English-made bromide is employed. This

* *Brit. Med. Jour.*, March 1[illegible], 1908.

W1
H069X

pronouncement conflicts with general experience of the drug, but it is possible that the skin-lesions depend upon some common impurity with which bromides are liable to be contaminated. Whatever be the ultimate verdict upon this variant of an old cure, the interest aroused by it may help to crystallise opinion upon the nature of idiopathic epilepsy. We know that epilepsy may be a symptom of grave organic troubles, such as uræmia, general paralysis, and cortical lesions: if it can be shown that "idiopathic" epilepsy represents nothing more than a reflex discharge produced by the action of peripheral stimuli, and capable of suppression by drugs which lessen irritability, we may hope by the early application of the remedy in sufficient quantities to restore the necessary balance before the disease has become by perpetual iteration the "cerebral vice" which Dr. McCallum considers it to be.

